# Microbial control of intestinal innate immunity

**DOI:** 10.18632/oncotarget.4780

**Published:** 2015-07-03

**Authors:** Martina Brave, Dana J. Lukin, Sridhar Mani

**Affiliations:** Departments of Medicine and Genetics, The Albert Einstein College of Medicine, Bronx, NY, USA

**Keywords:** Immunology and Microbiology Section, Immune response, Immunity

Intestinal innate immunity, a critical component of the intestinal barrier to noxious agents, is a master regulator of intestinal health. Dysregulation of intestinal innate immune features are pathognomonic of disease states such as inflammation and cancer [1]. The implication of loss of intestinal barrier function to human health is broad and goes beyond oncogenesis [2, 3]. Thus, a complete understanding of the molecular pathways governing the regulation of innate immunity in the intestines is a prerequisite towards finding cures.

**Figure 1:**
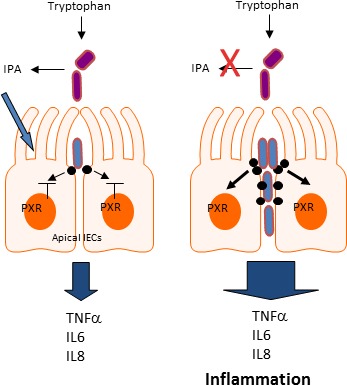
Proposed model of action of Tryptophan metabolite, Indole 3 Propionic Acid (IPA) in mice via PXR and TLR4 In this model, IPA binds to and regulates the intestinal barrier function through PXR. The latter receptor down-regulates TLR4 and reduces TNFα mediated intestinal barrier dysfunction. When IPA (e.g., antibiotic usage), or PXR is absent, TLR4 signaling is induced and there is aggravated inflammation and barrier dysfunction.

In a recent paper, our group defined a unique aspect of the regulation of intestinal innate immunity by microbial metabolites, in that, indole tryptophan metabolites (e.g., indole propionic acid) control intestinal barrier function via a host cellular pathway involving the Pregnane X Receptor (PXR) and Toll-like receptor (TLR-4) [4]. Based on the evidence presented, we speculate that the following model best describes this regulatory pathway (Figure 1). The model derives from data obtained from rodents (mice) and thus requires confirmation in humans. To better understand the general implications, however, some further discussion of available data from human and other rodent studies supports the foundations of our model.

In specific bacteria, with the availability of L-tryptophan, the repressed tryptophanase operon (*trp ABCDE*) and *tna* operon (*tnaCAB*), are induced, and indole concentrations rise [5]. Indoles generated in one species can cross cell membrane boundaries of permissive cells and participate in interkingdom signaling [5]. There is also a significant influence of the environment, other than the availability of L-tryptophan on TnaA expression (e.g., cell density, high pH, low glucose availability) [5]. The indoles have been implicated in interkingdom signaling among bacteria and in regulating (inhibiting) biofilm formation, motility, chemotaxis, and cell adherence. L-tryptophan supplementation of mice exposed to inflammatory toxins (DSS, TNBS) ameliorates inflammatory indices in the intestines and liver fat. Colitic mice have reduced indole metabolites [6]. In humans, urinary excretion of tryptophan is increased, and enhanced Indoleamine 2,3-dioxygenase (IDO) expression in inflamed enterocytes and rapid tryptophan catabolism in the intestines, results in low serum tryptophan and markedly increased serum kyneurine:tryptophan ratio [7]and end metabolites. In keeping with this concept, the anti-inflammatory shunting of tryptophan to serotonin/melatonin is blunted in patients with IBD. However, fecal tryptophan content is elevated suggesting a block in microbial tryptophan metabolism [8]. IPA is inversely related to systemic inflammation in overweight individuals as well as the increased IPA levels upon administration of anti-inflammatory dietary interventions to humans adds further proof of the importance of IPA in inflammation. Indeed, indole levels are significantly lower or absent in humans with alcohol-induced high intestinal permeability as compared to those with low intestinal permeability (personal communication, S Leclerq).

Microbial metabolites of tryptophan may have concentration dependent effects on the host. For example, as its anti-oxidant function, there could be deleterious effects when IPA is administered during active inflammation; however, when administered prior to active inflammation could have dose dependent protective effects in mice (unpublished data). High (non-physiologic) doses of IPA could indeed have toxic effects on intestinal organoids (unpublished data). Indeed, this could be further complicated by host metabolism (e.g., indoxyl sulphate) and such metabolites could have other consequences. These observations give us pause to be cautious in performing and interpreting experiments with microbial metabolites.

A related issue regarding our model is that the data generated focused on the small intestines. These studies were performed because the small intestines have very clear histologic architecture of the crypt and villus. However, our results are applicable to PXR expression and function in the large intestines. First, PXR is expressed in the colon. Second, we have shown an inverse relationship between PXR and TLR4 in human colonic samples obtained from health controls as well as from patients with IBD [4]. Human colon cancer cell lines show an inverse relationship between PXR and TLR4 [4]. Third, prior work by the Gonzalez laboratory (NIH) has demonstrated that PXR is a target for anti-inflammatory action in the colon. Finally, we have also shown that murine colonocytes exposed to a lipid A fraction (KDO2)(TLR4 agonist) in the presence of a PXR agonist (pregnane carbonitrile), results in reduced p38 phosphorylation in PXR wild-type mice but not in knockout mice (PhD Thesis, S. Mukherjee, 2013). Together, these results clearly show that the model is applicable to small as well as large intestines.

The mechanism of how PXR regulates TLR4 remains unknown. Indeed, the mechanism of binding of indoles and its metabolites to PXR also remains an active area of investigation. These studies will shed light on developing metabolite mimics as PXR activators and future strategies towards controlling breaches in intestinal barrier function.
